# The dark side of coproduction: do the costs outweigh the benefits for health research?

**DOI:** 10.1186/s12961-019-0432-3

**Published:** 2019-03-28

**Authors:** Kathryn Oliver, Anita Kothari, Nicholas Mays

**Affiliations:** 1 Department of Public Health, Environments and Society, Faculty of Public Health Policy, London School of Hygiene and Tropical Medicine, London, UK; 2 School of Health Studies, Western University, London, ON Canada; 3 Department of Health Services Research and Policy, Faculty of Public Health Policy, London School of Hygiene and Tropical Medicine, London, UK

**Keywords:** Coproduction, research ethics, stakeholder engagement, evidence use, policy and practice

## Abstract

**Background:**

Coproduction, a collaborative model of research that includes stakeholders in the research process, has been widely advocated as a means of facilitating research use and impact. We summarise the arguments in favour of coproduction, the different approaches to establishing coproductive work and their costs, and offer some advice as to when and how to consider coproduction.

**Debate:**

Despite the multiplicity of reasons and incentives to coproduce, there is little consensus about what coproduction is, why we do it, what effects we are trying to achieve, or the best coproduction techniques to achieve policy, practice or population health change. Furthermore, coproduction is not free risk or cost. Tensions can arise throughout coproduced research processes between the different interests involved. We identify five types of costs associated with coproduced research affecting the research itself, the research process, professional risks for researchers and stakeholders, personal risks for researchers and stakeholders, and risks to the wider cause of scholarship. Yet, these costs are rarely referred to in the literature, which generally calls for greater inclusion of stakeholders in research processes, focusing exclusively on potential positives. There are few tools to help researchers avoid or alleviate risks to themselves and their stakeholders.

**Conclusions:**

First, we recommend identifying specific motivations for coproduction and clarifying exactly which outcomes are required for whom for any particular piece of research. Second, we suggest selecting strategies specifically designed to enable these outcomes to be achieved, and properly evaluated. Finally, in the absence of strong evidence about the impact and process of coproduction, we advise a cautious approach to coproduction. This would involve conscious and reflective research practice, evaluation of how coproduced research practices change outcomes, and exploration of the costs and benefits of coproduction. We propose some preliminary advice to help decide when coproduction is likely to be more or less useful.

## Background

It is now widely recognised that research evidence requires ‘translation’, beyond the standard academic journal article into some format which can be easily absorbed by policy-makers, practitioners or other ‘users’, if it is to have influence outside the academy [1–3]. In addition to interventions to increase knowledge translation and uptake [4], there has been a re-evaluation of what ‘counts’ as evidence for practice and policy [5, 6]. Moving away from its medical roots, the evidence-informed policy and practice movement and debate increasingly value experiential and practical knowledge [7–12]. Many researchers also feel that their research skills should be deployed in the service of those members of society who are less empowered – through, for example, participatory action research [13] – and that science in general should be made more inclusive and democratic [14–16].

To achieve these diverse aims, collaborative research practices have been welcomed and promoted by many as the best way to answer these challenges [17–25]. There are many forms of collaborative research practices, including coproduction, co-design, co-creation, stakeholder and public engagement, participation/involvement and integrated knowledge translation all sit under this umbrella [25], reflecting a very diverse set of motivations, activities, identities and discourses about how research interacts with the rest of society [26]. Coproduction originally referred to the development of more responsive, personalised public services through “*the joint working of people who are not in the same organisation to produce goods or services*” [27–29]. These principles are increasingly applied to the production of knowledge, and coproduction is now a mainstream term in health research [19]. In addition, researchers are required and supported by universities, funders and publishers to create impactful research, with ‘engagement’ as a routine section on most grant proposals. Similarly, integrated knowledge translation approaches have gained traction, led by the example of Canadian researchers, governments and funders (e.g. Canadian Institutes of Health Research).

Given the confluence of ethical, practical and procedural reasons in support of coproduction, one might think that there is a good level of consensus about what coproduction is, how to do it, what the effects are, and an evidence-based set of techniques to achieve these effects. In fact, there are very few evaluations of how coproduction works in practice or on the impact of coproduction on research, practice, policy or population outcomes [30]. Given the pressures on researchers to coproduce in order to create research impact, we feel it is timely to closely examine the arguments and evidence for this. The purpose of this paper is to present the motivations for coproduction, identify associated tensions and costs, and identify where coproduction appears warranted.

### Why coproduce?

There are four main arguments for coproduction in the literature:

1. **Substantive:** where engagement is undertaken to improve the quality of the research [31]. This may be through helping researchers and policy-makers develop a more holistic understanding of a context, an issue and/or a solution [32, 33], both epistemologically and ontologically [25, 34]. The mechanisms by which research quality may improve are not always articulated, but many commentators agree that engagement makes research more relevant by focusing on appropriate topics which need elucidating [24, 35], and that by talking with others, we discover ‘unknown unknowns’ [36] and create new knowledge together.
2. **Instrumental:** these arguments are based on “*a desire to see research findings utilized in effective ways*” ([31], p. 230). Many commentators claim that co-designed and coproduced research is likely to be more impactful [23, 24, 35, 37]. The actual process of collaborative research can help identify practice-based research questions and outcomes that are related to the implementation setting [38]. This includes practical mechanisms such as upskilling and creating capacity amongst non-academics [29, 35], and creating a sense of trust and empowerment amongst potential users, thus increasing the likelihood of research utilisation [39] and of evidence sharing [40, 41]. Innovative research designs and feasible ways to collect data can be identified through collaboration [42]. Further, stakeholders can describe how receptive the practice or policy setting is for subsequent implementation of the research findings [43].
3. **Normative:** where engagement is simply “*of intrinsic value*” ([44], p. 153). This set of arguments connects to the discourse of the civic or public university/academic, focusing on accountability to (public) funders, and the conduct of research to serve public interests. Second, authors argue that sharing expertise (in its guise as power) is simply a way to be fairer and more ethical [37, 45]. Flinders characterises this view as the belief that coproduction can be “*transformative not solely in research terms but in social terms: the engagement of citizens and social groups nourishes the renewal of democracy*” ([26], p. 261). This often sits alongside a view that mutual and continual learning is a virtue of collaborative research practices – a clear shift from the paternalistic ‘science advice’ model still prevalent (see e.g. [46, 47]). Of course, individuals may also view coproduction as a satisfying and enjoyable way to practise – a rarely articulated but, in our view, valid justification for attempting it.
4. **Political:** Often less explicitly made, but equally important, is a set of arguments used to justify coproduction on political grounds. By involving non-researchers in the elite, de facto exclusive process of research, coproduction can make users feel empowered and included [48, 49], and increase a sense of ownership (a prerequisite for acting on research findings) [39]. The integrated knowledge translation view specifies partnering with research users who have the authority to implement the findings [50]. Greater collaboration can reduce negative stereotypes held by researchers and policy-makers about each other [51], and improve trust [36, 52], smoothing the way for research to be more impactful. Several commentators also claim that coproduced research is more credible and relevant to intended audiences [39], and that the legitimacy of the knowledge [53], goals and activities [54] are increased. The motivation for either approach – collaborating with the disempowered or the powerful – is political, in the sense that it is about changing the attitudes of different groups to attain a particular end, even to the extent of being explicitly framed as a political and strategic response to diminishing belief in public services [55].

Coproductive approaches are therefore used for a variety of reasons. In the spirit of evidence-based enquiry, we ask whether coproduction is likely to enable researchers to realise these four objectives and at what cost.

### How to coproduce?

There is a multiplicity of modes by which researchers may interact with stakeholders [25]. At very low, shallow levels of involvement, stakeholders may be the mere recipients of research findings [56]. More active interaction types may include reaching out through soft interpersonal networks to seek advice about policy agendas, or to influence policy dialogues individually [57]. Researchers may be employed by public policy institutes to do research commissioned by and directly applicable to policy issues. At the most engaged level, what many would regard as ‘truly’ coproductive approaches have been tried in specific, partnered project grant calls, research institutes or long-standing research programmes (such as the English NHS Collaborations for Leadership in Applied Health Care and Research; CLAHRCs) [58–61] implying an equality of status between all co-researchers. Often, a financial contribution by the stakeholder is indicative of an authentic coproduction partnership. These coproductive approaches often include elements such as provision of opportunities to co-learn about research and substantive topics, to work iteratively over the lifespan of a project to co-steer the questions and direction of research, or to develop interpretations of data and implications of these together. However, there are very few evaluations of such approaches or projects, and existing evaluations often point to a “*lack of hard evidence as to its impact on the quality of research*” ([62], p. 3) and a need to “*determin*[e] *best practices for engaging patients*” ([62], p. 3). We concur with these findings.

As originally intended, coproduction referred to a significant shift of power from researchers or decision-makers to service users (e.g. patients) [26, 27], implying deeply embedded collaborative practices. However, it has come to refer to activities as diverse as consultation on topics, co-designing research questions, co-interpreting results and recommendations, and embedded or in-house researchers within policy or service delivery agencies [25]. These activities describe very different modes of interaction, and imply different practical resourcing needs, skills and processes, as well as outcomes. However, there is very limited evidence about the impact of each type of strategy [25, 60, 63], no consensus about the best theories to use to inform this work [64], and little knowledge as to which strategies will help achieve which aims. Yet, millions of pounds are invested into deliberative, coproductive, collaborative research processes, and to related initiatives supporting research uptake and research impact, without (1) looking at what works or (2) knowing the pros and cons of different approaches or (3) evaluating and monitoring these processes. We might expect to see changes to policy or practice, to population outcomes, to research practices or to research outputs, yet no evaluation of coproduction attempts to explore all the domains holistically [65, 66]; this matters, because coproduction is not free of risk or cost. As Flinders et al. note [26], coproduction carries significant risks for academics, who are required to adopt practices far from those traditionally taught, adopted, recognised or rewarded by the academy. They argue that coproduction is “*time-consuming, ethically complex, emotionally demanding, inherently unstable, vulnerable to external shocks, subject to competing demands and expectation*” ([26], p. 266). In the next section, we explore how coproductive approaches may generate tensions through the research process and identify costs to participants.

### Challenges and costs to coproduction

Challenges can arise throughout the lifecycle of a research project, and more generally through the research process (see Table 1 for a non-exhaustive list based on our own experiences). From the framing of research questions to the development and dissemination of recommendations, coproductive research can cause conflict, consume resources and lead to misunderstandings. The very purpose of a collaboration may not always be clear to all, or be shared. The direction of a research project may not (or possibly cannot) reflect the values and priorities of all stakeholders, and team members may have different views about what the research findings show, or what to do with them.

**Table 1:** Challenges and costs in coproduced research

	Challenges which may arise	Costs
Developing mixed research teams	Stakeholders not homogenous, and can disagree ‘Usual suspects’ can take over, where coproductive discussions are dominated by certain individuals	The research process may take more time compared to a traditional research process Shared decision-making is threatened when process dominated by certain voices or interests
Framing research questions	Stakeholders and researchers may have different priorities and values Useful research can lack originality Research can be co-opted by partners, for example, to justify status quo or historical decisions	Damage to interpersonal or organisational relationships Damage to research careers Damage to researcher independence and credibility
Collecting data	Researchers may pressure stakeholders to allow their organisational resources to be used to facilitate data collection –e.g. using staff time or applying pressure for site access	Damage to interpersonal or organisational relationships, particularly with more powerful stakeholders
Analysing and interpreting data	Stakeholders may want to know which participant agreed to participate or what they contributed to the dataset Stakeholders may want to help analyse the data	Violation of research ethics obligations Researcher needs to train stakeholders and format data in an appropriate way to conform with research ethics obligations
Formulating recommendations	May be little agreement about the importance of research Researchers may be pressed to frame findings in particular ways	Findings are misrepresented Damage to researcher independence and credibility
Disseminating research	Researchers or stakeholders may be prevented from sharing unwanted findings Stakeholders may want to share findings before researchers are ready	Damage to researcher independence and credibility Damage to the credibility of the research process
Implementing change	Tension between advocating for research, or advocating for policy/practice changes Researchers show little interest in providing assistance with implementation efforts	Can damage relationship with practice or policy colleagues Implementation of research findings fail

**Fig. 1:**
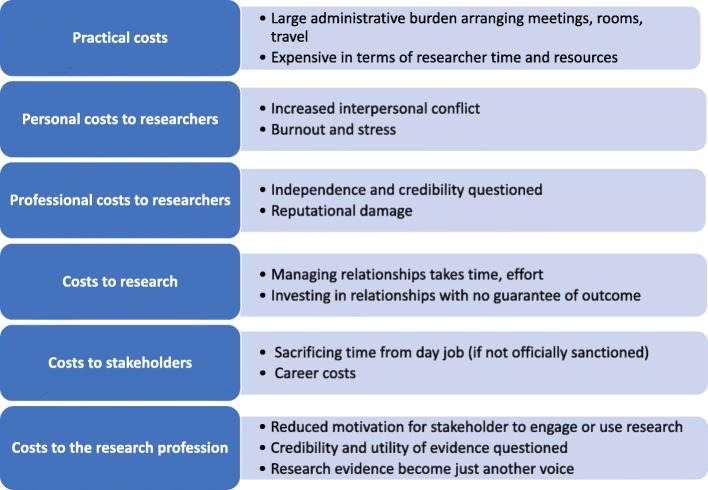
Costs of coproduction

Beyond the lifecycle of an individual research project, coproductive research can also mean (1) investing time and resources into relationships with no guaranteed concrete output; (2) calling on favours and being able to provide favours in return; and (3) in general, having the capacity to manage sometimes tense relationships. Each of these challenges is also associated with costs for researchers and stakeholders. Below, we list and describe these costs (see Fig. 1 for a summary).

Firstly, there are clear practical costs to doing research in this way. It is expensive, as it requires the presence or time of multiple actors who are often not on site, have other primary responsibilities, or need travel or other reimbursement. The administrative burden of arranging spaces and attendance is significant. In addition, it takes skill to recruit and manage mixed groups who are working together to create something new. Managing group dynamics, not letting the loudest dominate and maintaining rules of conduct require interpersonal skills which not all academics are trained in or endowed with. Thus, a set of professional skills and resources are needed – but this type of work is often added on (to ‘real research’) with little thought for how to properly resource it [63]. A gendered aspect may feature in coproduction work; our own experiences suggest that coproduction conferences are attended by women and coproduced projects are often led by women. Senior men may be keener to manage coproduction work with senior managers, clinicians and policy advisers than with lay people, patients, carers, etc. How, why and the consequences of this possible imbalance require further thought.

Second, there are significant personal costs to researchers. Many of the tensions above play out through interpersonal conflict, difficult conversations and/or outright disagreement. Many people find this very difficult to handle in the workplace, and when research careers, outputs and funding are at stake, this only adds to the pressure. There are reputational risks associated with conflict, which, for many, is reason enough to avoid difficulties; yet, ignoring these challenges and going along with stakeholder views with little negotiation of mutual interests can lead to unnecessary duplicative research and perhaps less imaginative or derivative research, which can also compromise research(er) integrity. It is too easy to dismiss these tensions as being because of interpersonal tensions; rather, we argue that these tensions are how inherent power imbalances and conflicts in coproductive research are expressed. Badging them as being because of interpersonal difficulties dismisses the emotional labour of working collaboratively, and risks ignoring the need to find solutions for real challenges inherent in this type of research practice. Managing the engagement process takes personal qualities that are not hugely abundant amongst the academic population. These interpersonal conflicts can lead to – as can the additional work associated with coproduction – serious stress and burnout [67].

Third, there are professional costs to researchers. Taking time out of seeking research funding, writing publications in impactful journals, doing administration, and teaching is a huge ask for academics, and coproduction requires significant commitment of time and resources, often with little guarantee of success or recognition. Coproduction can lead to research outputs that are regarded as being of lower quality than ‘real’ or ‘pure’ research [26], or simply hard to publish. For engagement to occur successfully, researchers are often required to do professional favours (e.g. do extra presentations), be easily and readily available, and be able to engage with no expectation of a guaranteed impact as measured in academia. This is clearly not the case for all researchers, and may lead to the costs of coproduction being borne by those least able in terms of time or capacity – in our experience, often the most junior, temporary, female and/or of colour.

Additionally, researchers risk being seen as partisan and/or lacking in credibility [68, 69], by both their colleagues and by their stakeholders. Researchers may find they are being used to add legitimacy to political positions [70] and may find that adopting political or policy positions, or being seen to endorse political viewpoints, can hinder their ability to work with a range of stakeholders – even compromising personal safety [71]. It can also lead to the view that the research itself is not a neutral representation of the data, but a cherry-picked narrative to support a political position – producing “*policy-based evidence*” [72]. Finally, as well as being regarded as partisan and biased, coproductive researchers risk being regarded as an academic “*lightweight*” [73], producing nothing of substance.

Fourth, there are risks to the research itself. Under business-as-usual rules, researchers spend their time identifying genuine and novel gaps in the knowledge base, which have to be justified at length to colleagues and funders. However, the coproduction process can lead to researchers being asked to answer questions which are dull, not novel (little contribution to the scientific literature), or not generalisable (focused on local issues) – and therefore not easily publishable. As Kothari indicates, coproductive research can “*encourage a reassertion of control and power by dominant individuals and groups*” who possess the skills to engage with the project, or those whose contributions fit in with any preconceived ideas of the ‘right’ responses will be highlighted over those who “*lack the skills to perform as required*” ([74]: p. 142). This can lead to research being conducted on unnecessary or repetitious topics, or on topics which serve the interests of some groups over others. Of course, this is often the case, but coproduction offers a vision of equality and fairness which can mask these dynamics.

The research process can be significantly affected, with delays caused by the additional work of recruiting and engaging with stakeholders; differences of opinion about the purpose and role of coproduction or the research itself; tensions around how to frame interpretations and recommendations that may result in research being co-opted or researchers being silenced (at a micro level, during conversations, or procedurally through being prevented from publishing); and in how and when to share the findings. These concerns are not new to implementation science or ethnographic researchers (see, for example [75–77]) and the tensions around the research process itself have long been a domain of inquiry for social researchers. Yet, in the current climate, the culture of hit-and-run research (get funding, do research, achieve impact, leave) does not easily allow researchers the time and space to reflect on their practice, and any proffered thoughts would be considered incidental to the main project, irrelevant or possibly unwanted by funders.

Fifth, there are costs to stakeholders. In addition to taking time and resources away from their primary responsibilities, coproduction requires that participants – especially policy-makers – make themselves vulnerable, by sharing uncertainties or sensitive information [36]. This could include admitting the political uncertainty, lack of policy direction, or political mistakes – all of which could incur costs. More broadly, one can imagine scenarios where participants were asked for input, which was then cherry-picked to suit research agendas, or where their contributions were not treated transparently and systematically. Policy-makers, service users and professionals can feel ignored or used, may not understand why their voices are not represented in the way they had expected, or may feel that they were not accorded the usual respect they enjoy in the workplace. Far from empowering people politically, participation in research can lead to personal narratives and experiences being dominated by senior interests, leading to lack of motivation to engage again [54], and the ‘subjugation’ of participants [78, 79].

Finally, there are costs to the entire profession, or the ‘scientific endeavour’, by the reduction of trust in science and scientists. Doing coproduction recklessly, discourteously or without due attention to professional etiquette can cause significant ill-feeling about participating in research. This can lead to mistrust of researchers, and a lack of willingness to engage in other research processes. Furthermore, the issues about independence and credibility can call the value of academic research into question. If researchers are working so closely with parties with clearly vested interests in particular aims, how can anyone be confident that the findings have been represented truly and holistically? This can lead to research being viewed as simply one more lobby group, rather than holding a special status [80].

### A cautious approach to coproduction: how to minimise the costs and maximise the benefits of coproduction

We recognise the transformative potential of coproduced research, and also feel that calls to do impactful research are unlikely to go away. If done well, coproduced research processes can – indeed must – manage and alleviate these tensions. Yet, there is a significant dearth of empirical evidence about coproduction processes or outcomes, which makes its widespread advocacy troubling. We simply do not know which strategies are the most promising for collaborative research. For example, which stages of the research process are critical for stakeholder involvement, and which stages are less essential? What are the best ways to keep stakeholders engaged with the process? Under what circumstances should coproduction be used, rather than other (e.g. consultative) approaches? What types of infrastructure need to be in place to support productive coproduction?

What then should be done? We suggest a two-step process when commissioning and/or initiating each research project, as follows:

- First, considering whether coproduction is likely to be useful in helping the research meet its aims and selecting strategies accordingly, and
- Second, considering whether other approaches are as or more likely to help achieve those aims.

This process is likely to be the responsibility not just of the researchers but also policy-makers, practitioners, funders and patient/user representatives.

Using the four arguments for coproduction above, researchers and others (that is, funders, policy-makers, managers, clinicians, the public, patients, carers, etc., depending on the topic) should identify specific potential aims for any coproduction process – for example, to identify or refine a research question, or to broker access to a political community – and decide whether coproductive strategies can best help them achieve those aims, and if so which ones. Having identified the purpose of the research and its desired outcomes in relation to the context and the interests potentially affected by the findings, the researchers and others should attempt to identify the best strategies to achieve these, and thus decide when and where coproduction might help, and when and where it might not.

Given the lack of much evidence in this area, we have to make judgements based on the circumstances of each project. Some early thinking along these lines is summarised in Table 2, which particularly relates to coproduction of evaluations with policy-makers and service managers.

**Table 2:** Some early thoughts on when to favour coproduction and when not to

Less emphasis on coproduction when …	More emphasis when …
The policy or programme is likely to be controversial or the findings are likely to be contested	The policy or programme is widely regarded as a ‘good thing’ and the findings are unlikely to be contested
Conflicts of interest between stakeholders are likely to be hard to manage (e.g. policy-makers are directly responsible for the successful delivery of a policy or programme)	There are few fundamental conflicts of interest between stakeholders (e.g. policy-makers are not directly responsible for successful delivery of a policy or programme)
There is less concern to use the findings directly and immediately for policy or management decisions	The main goal is to ‘use’ the findings for policy and management decisions
Funders and/or commissioners of the research value ‘expert’, dispassionate scientific inquiry above other forms of knowledge	There are few concerns about the limitations of ‘policy-based evidence’
The nature and purpose of the policy or programme is relatively well defined and agreed upon	The policy or programme still needs considerable definition, refinement, testing of feasibility, acceptability, etc.
The prime purpose of the research is to establish whether the policy or programme ‘works’ and there is strong prior commitment by policy-makers or managers to acting on the findings (i.e. ‘decision space’ is available)	The prime purpose of the research is to identify how best to implement the policy or programme rather than whether or not to proceed with it
Undertaking the research is less dependent on cooperation of policy agencies or local programme implementers	The research cannot easily be carried out without the active cooperation of policy-makers and/or local programme implementers
There are good, informal, ongoing relationships between the researchers, and policy-makers and service managers	There is a need to increase mutual awareness and understanding between researchers, and policy-makers and service managers
One or more of time, resources and expertise are in limited supply to involve the key stakeholders at all appropriate points in the research process	Time, resources and expertise are available to involve the key stakeholders at all appropriate points in the research process

We suggest that a cautious approach to coproduction would include examining the costs and benefits to all involved, recognising the significant costs and risks to investing time and resources into good facilitation and management of expectations, establishing ground rules and processes, and deciding on evidence-informed strategies to achieve established and shared aims and outcomes. Below, we summarise some key questions which we believe all those involved in coproduction should ask themselves prior to attempting any engagement.

Individual researchers, funders, commissioners and participants in the research process should ask themselves:

1. What is everyone bringing to the table? For example, policy-makers and funders bring money, knowledge of the political context, pressure for answers; researchers bring topic and methodological expertise; public and patients bring their experiences.
2. Under which circumstances are these needed, for what purpose, and at which stage of the research process? For example, when is it better to have patient representatives articulate the user perspective rather than derive understanding from a systematic review of patient experiences? Should policy-makers be involved all the way through, or only at specific stages?
3. What are the costs? How will the time, administrative, cultural and professional costs be borne and defrayed by those involved?
4. How are decisions taken, and how will responsibility and accountability be shared? Will group dynamics, market forces, authority or some other decision-making process control the process? How will this be governed and managed?

Research organisations and funders need to consider:

1. How to create (co-create) and support the infrastructure and leadership for coproduction
2. How to provide training in coproduction, and help researchers and funders take this seriously as a skill set
3. How to reward good practice, and to recognise the work coproduction may take even if it does not lead to impact
4. How to evaluate the potential impact(s) of coproduction
5. How to ensure that coproduction supports, rather than mitigates against diversity and quality in research and policy

## Conclusions

Coproduction is an exciting approach to research that can generate truly novel, unexpected outputs [19]. However, it takes investment, skills, time and courtesy. Engaging stakeholders should be done for the right reasons and in the right way [52], yet there is so little empirical evidence about how coproduction changes research, policy or practice, or how it may compare to alternatives. Coproduction as a way to generate knowledge can be seen as one way to make the resulting research more ‘implementation ready’ on the assumption that, through the coproduction process, the team has already anticipated research users’ needs, capacities and priorities. Yet, there may be alternative ways to achieve this outcome without risking the costs detailed above.

The main motivations of researchers to engage in coproductive work are of course positive, with genuine belief in the importance and potential transformational nature of working with those outside academia. However, the political arguments are often not made explicitly (see, e.g. [31]). To us, this suggests that, either some researchers engage naively (albeit in good faith), or that researchers appreciate the political nature of decision-making and research, but prefer not to openly and honestly explain their strategies to deal with these dynamics. We also note that most normative, substantive and instrumental justifications for coproduction can be achieved through other means. Thus, we argue that political reasons for engaging in coproductive research may be the least-discussed, yet most important rationale made by researchers. Given the costs, we argue that this requires serious attention by the research community, including those who commission, participate in and evaluate research.

Coproduction and participation may have a profound impact on the practice of research and the process of decision-making, and there are many promising and exciting examples of joint inquiry between researchers and stakeholders [30, 59, 81–83]. There are certainly many unanswered questions about the potential value of coproduction and collaborative research, and outlining the benefits and the mechanisms generating those benefits is an important task.

However, the tensions and challenges, costs and opportunities must also be described; they are unequally experienced and borne, strongly suggesting that mindful engagement is essential for the ethical practice of research. Many of these points of tension and costs are about power, and we recognise that all the implementation type tools, frameworks and processes cannot design away these tensions. There must be a more reflective, open discussion about when to do coproduction, the ethics of coproduction, and so on; we need more empirical evidence about its processes and outcomes; and we do not know which types are best suited for which problems, institutions or team configurations. As a community, it is imperative that we are more reflective about how coproduction influences the process of research, and the roles and responsibilities of everyone involved in collaborations [84].
